# Development of a Methodology for Measuring Oxytocin in Feces: Insights from a Preliminary Study in Captive Lions (*Panthera leo*)

**DOI:** 10.3390/ani15162409

**Published:** 2025-08-17

**Authors:** Paula Serres-Corral, Vanessa Almagro, Loles Carbonell, Santiago Borragán, Eva Martínez-Nevado, Miguel Angel Quevedo, Hugo Fernández-Bellon, Annaïs Carbajal, Manel López-Béjar

**Affiliations:** 1 Department of Animal Health and Anatomy, Universitat Autònoma de Barcelona, 08193 Bellaterra (Cerdanyola del Vallès), Spain; anais.carbajal@uab.cat; 2 Parc Zoològic de Barcelona, 08003 Barcelona, Spain; 3 Bioparc Valencia, 46015 Valencia, Spain; 4 Parque de la Naturaleza de Cabárceno, 39690 Obregón (Villaescusa), Spain; 5 Zoo Aquarium de Madrid, 28011 Madrid, Spain; 6 Zoobotánico Jerez, 11408 Jerez de la Frontera, Spain

**Keywords:** fecal oxytocin, peptide hormone, non-invasive, extraction, enzyme immunoassay, animal welfare, wildlife

## Abstract

Oxytocin (OT) has emerged as a central focus in stress regulation and animal welfare research. While commonly measured in blood, saliva, and urine, its presence in feces remains unexplored. Fecal OT (fOT) could be highly valuable for wildlife studies, where direct handling is impractical. This study explored OT detection in feces using lions (*Panthera leo*) as a model species. The objectives included developing a reliable fOT extraction protocol, describing baseline fOT patterns in captive lions, and evaluating its relationship with fecal glucocorticoid metabolites (fGM) under non-stressful conditions. Fecal samples from 16 lions in stable social groups across five Spanish zoos were collected over two to six weeks. A methanol-based extraction and commercial enzyme immunoassay were successfully validated for fOT quantification. Concentrations ranged from 3.00 to 296.64 ng OT/g of feces, with notable intra- and inter-individual variability. Age, sex, and contraceptive status had no significant influence on fOT (*p* > 0.05), but concentrations differed significantly between zoos (*p* < 0.001). No consistent relationship was found between fOT and fGM levels under baseline conditions. This study is the first to demonstrate OT detection in feces. The validated methodology provides a foundation for investigating fOT as a biomarker of stress and welfare in wild and domestic species.

## Simple Summary

Oxytocin is a hormone involved in social bonding and stress regulation which has attracted great interest in animal welfare research. However, it has never before been measured in feces, a method that would allow for non-invasive monitoring. This study investigated whether oxytocin can be consistently detected in fecal samples, using lions as a model species. The aims were to develop a reliable method for extracting and measuring fecal oxytocin, to describe typical patterns of this hormone in captive lions, and to examine its relationship with glucocorticoid metabolite concentrations in feces. Samples were collected from sixteen lions across five Spanish zoos. Oxytocin was successfully measured in feces with the extraction method developed, with considerable variation among individuals and zoos. Age, sex, and contraceptive status did not significantly influence oxytocin levels, but concentrations differed between zoos. No consistent pattern was found between oxytocin and fecal glucocorticoid metabolites under stable housing and social conditions. These findings provide initial evidence that oxytocin can be measured in feces, although several methodological aspects remain to be investigated. Overall, this non-invasive approach shows promise as a tool for assessing stress and welfare in both wild and domestic animals.

## 1. Introduction

Oxytocin (OT), a nonapeptide synthesized in the hypothalamus and released into circulation by the neurohypophysis, was initially recognized for its roles in parturition and lactation [1,2,3]. Beyond these peripheral actions, OT also acts as a neurotransmitter within the brain, influencing behavioral and neuroendocrine functions [4]. In recent decades, OT has garnered significant attention for its involvement in social behavior, as it is critically involved in social bonding and maternal behavior [5,6]. Furthermore, this neurohormone has also been shown to modulate stress responses partly through direct inhibitory actions on the hypothalamic–pituitary–adrenal (HPA) axis [4,7]. More recently, OT has become a focus in animal welfare research due to its dual role in regulating stress and promoting positive affective states [8,9,10]. While traditional welfare assessments often focus on negative affective states, current trends advocate for incorporating indicators of positive welfare [11,12]. Accordingly, research efforts are now directed towards developing and applying novel biomarkers indicative of positive affect that, combined with traditional indicators such as glucocorticoids (GCs), offer a more comprehensive assessment of an animal’s well-being [13,14]. In this context, OT has been proposed as a biomarker of positive affective states due to its role in facilitating prosocial behaviors and regulating stress [9,10].

Despite its promise, the oxytocinergic system remains complex and not fully understood. Recent findings suggest that OT is produced not only in the hypothalamus but in peripheral tissues, such as the gastrointestinal tract [1,15,16]. Furthermore, the release patterns of hypothalamic OT within the brain and into the peripheral circulation may differ depending on the context or stimulus [17]. Likewise, little is known about OT excretion patterns, including potential circadian or seasonal rhythmicity. While some studies suggest a circadian rhythm in cerebrospinal fluid OT levels in humans and non-human primates, no consistent patterns have been reported in plasma or peripheral matrices, and the findings remain mixed across species and sample types [18,19]. This complexity raises questions about whether peripheral OT levels accurately reflect central activity [20,21,22] and the biological relevance of OT measurements across biological matrices [9,23].

Due to the invasiveness of measuring central OT (i.e., in cerebrospinal fluid), most behavioral and welfare studies rely on measuring OT levels in peripheral matrices, such as blood, urine, or saliva [23]. Although most studies to date are based on the measurement of OT in serum or plasma, blood collection remains an invasive procedure in most species, particularly wildlife, and the sampling itself can interfere with the results of behavioral and stress-related studies [24]. Consequently, non-invasive matrices, such as saliva and urine, are increasingly being validated and applied for the measurement of OT in both domestic and wild species (i.e., urine in dogs (*Canis lupus familiaris*) and wolves (*Canis lupus*) [25], cats (*Felis catus*) [26], baboons (*Papio ursinus*) [27], chimpanzees (*Pan troglodytes*) [28], western lowland gorillas (*Gorilla gorilla gorilla*) [19], or African elephants (*Loxodonta africana*) [29]; and saliva in dogs [30], several other domestic species [24,31], or gorillas [19]). More recently, OT has also been measured in the hair of pigs [32] and in the sweat of humans [33], expanding the range of matrices in which this neurohormone can be investigated. Even though these less invasive matrices are progressively being validated for the measurement of OT across domestic and wild species, the measurement of OT in feces remains unexplored.

Unlike other matrices, feces can be easily collected without disturbing the studied animals, allowing regular sampling over long periods of time [34,35,36]. In captive and free-ranging wild animals, where capture and handling is often not feasible, measuring fecal OT (fOT) would prove most valuable. Fecal hormone monitoring is already well-established for steroid hormones, and is widely applied to evaluate stress and reproductive endocrinology in animals [34]. However, while the metabolism and excretion pathways of steroids into feces are well-documented [37], this is not the case for OT. Peripheral OT is eliminated from circulation by the liver and kidneys [38,39,40] and, although some studies have investigated OT clearance in urine [41,42,43], research on OT excretion through feces is lacking.

Before investigating the potential role of this peptide hormone in feces in relation to social behavior and stress-regulation, a crucial first step lies in developing and validating a reliable methodology for the measurement of fOT. Accordingly, in the present study we aimed to establish for the first time a methodology for extracting OT from feces that could be broadly applied to both domestic and wild animal species. To accomplish that, we first compared two commonly used organic solvents for fecal hormone extraction and selected the one with best extraction efficiency and assay performance. We then analytically validated a commercially available enzyme immunoassay (EIA) for the quantification of fOT in lions (*Panthera leo*) as a model species. Although the commercial kit selected is not specifically designed for fecal samples, it has been previously used and validated for the measurement of OT in non-conventional matrices. Following this validation, we explored patterns of fOT excretion in captive lions under stable social and environmental conditions, and evaluated the relationship between fOT and fecal glucocorticoid metabolite (fGM) levels in captive lions under baseline conditions.

## 2. Material and Methods

### 2.1. Study Animals and Sample Collection

Fecal samples from a previous study on steroid hormones in captive lions [44] were utilized to develop and validate a methodology for extracting fOT. These fecal samples were collected opportunistically from 16 lions (10 females, 6 males; mean ± SD age: 10.1 ± 4.2 years old) housed at five Spanish zoological institutions: Barcelona Zoo (BCN), Bioparc Valencia (BPV), Parque de la Naturaleza de Cabárceno (CA), Zoobotánico Jerez (JZ), and Zoo Aquarium de Madrid (MAD). Among lionesses, the contraceptive status varied by zoo and included intact females, hormonally contracepted females (with deslorelin acetate implants, SUPRELORIN^®^, Virbac, France), and ovariectomized females (see Section 3.4 for individual biological data including age, sex, and contraceptive status). All animals involved in this study were housed and managed as usual by the zoological institutions, with no experimental manipulation or intervention.

Outdoor enclosure sizes varied notably across institutions, ranging from 150 m^2^ at JZ to 16,000 m^2^ at CA. Intermediate enclosure sizes included 360 m^2^ at BPV, 1090 m^2^ at BCN, and 1600 m^2^ at MAD. The feeding routine generally consisted of evening feeding and included some form of dietary restriction once a week, typically fasting (e.g., BCN, JZ, MAD) or a reduced ration (CA), except for BPV, where animals were fed daily. Climatic conditions across all five zoos are temperate Mediterranean, with mild, wet winters and hot, dry summers.

At the time of sampling, all lions were in good health and maintained in stable social and environmental conditions. The sampling took place for two to six weeks between March and April 2019 (BCN, 3 weeks) and between April and June 2021 (BPV, 3 weeks; CA, 6 weeks; JZ, 2 weeks; and MAD, 3 weeks). The sampling effort therefore varied slightly across zoos. Samples were collected daily during the morning cleaning routines and were stored at −20 °C. Non-toxic shredded colored waxes (GIOTTO Be-Bé^®^, Fila Iberia S.L., Barcelona, Spain) were used as fecal markers [45] in those zoos in which individual identification of the feces was not possible with the usual management practices (i.e., individual housing). Briefly, each lion was assigned a specific color, and a small portion of meat covered with shredded wax crayon of that color was offered to them individually each morning before routine feeding. Subsequently, macroscopic examination of the samples was conducted to identify the presence of the shredded wax crayon and to ascribe each sample to the proper individual.

### 2.2. Selection of Solvents and Extraction Procedure

Two organic solvents commonly used to extract different types of hormones were selected and independently tested to determine which was more effective for the extraction of OT in feces: methanol (MeOH), widely used for steroid hormone extraction [37,46], and acetonitrile (ACN), used for the extraction of peptides and small proteins [47,48]. Both solvents have been employed for the extraction of OT in other matrices (e.g., ACN in plasma and saliva [30,49]; ACN and MeOH in hair and urine [32,50]). Each solvent was tested separately, and we compared their extraction efficiency and performance in the subsequent validation tests.

We determined the most suitable organic solvent percentage for OT extraction in feces based on the previous work by Gnanadesikan et al. (2022) in urine [50]. This study concluded that, in their respective extraction protocols, 50% MeOH and 30% ACN were optimal for eluting OT without significant interference from other molecules present in the matrix. In our study, we used 55% MeOH for the initial extractions instead of 50%, as this percentage is already employed in the validated protocol for steroid extraction from lion feces [45]. This approach was intended to simplify the laboratory workflow, allowing for a single extraction procedure to be used for both steroid hormones and OT, in case MeOH proved to be the more effective solvent.

The extraction of OT from feces was based on the procedure commonly used for steroid extraction in feces [46]. Specifically, the protocol developed was based on the one routinely used in our lab for the extraction of steroid hormones from lion feces [45]. Briefly, fecal samples were dried at 60 °C in an oven (Memmert Universal oven UF55, Memmert GmbH, Schwabach, Germany) for four to six days. Once completely dry, samples were ground using a Mixer Mill (MM2 type; Retsch GmbH, Haan, Germany) and sieved with a mesh strainer to remove undigested and/or fibrous materials. Then, 300 mg of the sample were weighted, and 5.5 mL of the corresponding solvent (55% MeOH or 30% ACN) were added to the mixture. Samples were vortexed (Vortex Mixer S0200-230 V-EU; Labnet International Inc., Edison, NJ, USA) for 30 min and then centrifuged (Hermle Z300K; Hermle Labortechnik GmbH, Wehingen, Germany) at 7750× *g* for 15 min at 23 °C. The supernatant was transferred into 1.5 mL microcentrifuge tubes and the fecal extract was frozen at −20 °C until analysis.

### 2.3. Extraction Efficiency of MeOH and ACN

To determine the best solvent for extracting fOT, four pools of fecal samples were generated (Pool 1 and 2: females; Pool 3 and 4: males). Each pool consisted of 10 fecal samples obtained from at least 3 lions that had been previously dried and ground. The pulverized fecal samples were mixed together and further homogenized using a Mixer Mill (MM2 type; Retsch GmbH, Haan, Germany). Aliquots of each pool were freshly and independently extracted in parallel using either 55% MeOH or 30% ACN, as described below.

The efficiency of the extraction procedure for each solvent was determined by spiking standard solution of purified OT (ref #331-21292, Raybiotech Inc., Peachtree Corners, GA, USA) into the powdered sample pools before extraction. For each solvent (55% MeOH and 30% ACN), three aliquots (300 mg) of each of the four pools were spiked with 0.1 mL of high OT standard (820 pg/mL), low OT standard (370 pg/mL), or buffer solution (unspiked control), and 5.4 mL of the corresponding solvent solution (55% MeOH or 30% ACN) were added. Subsequently, extraction on the samples was performed as previously described (Section 2.2).

The extraction efficiency recovery percentage (*%REE*) was calculated using the values of the measured concentration of the *spiked pools* (high or low OT concentration), the measured concentration of the *unspiked control*, and the measured concentration of the *OT standard* with the following formula:

$$\%REE=\;\frac{Spiked\;pool\;-\;Unspiked\;control}{OT\;standard}\;\times 100$$

### 2.4. Oxytocin Assay Validation

Fecal OT concentrations were measured using the DetectX^®^ Oxytocin EIA kit (Product #K048-H; Arbor Assays, Ann Arbor, MI, USA). The cross-reactivity reported by the manufacturer is as follows: oxytocin 100%, isotocin 94.3%, mesotocin 88.4%, lys8-vasopressin 0.14%, arg8-vasotocin 0.13%, and arg8-vasopressin 0.12%. We selected this commercial kit as the assay anti-OT antibody had been rigorously characterized beforehand. In their study, Gnanadesikan et al. [50] demonstrated that the Arbor Assays antibody primarily recognizes OT through residues 7–9, with the OT conformational structure being most irrelevant for antibody binding. In addition, they found that three biologically active OT metabolites cross-react with the antibody, but cross-reactivity decreases substantially for the smaller fragments.

The performance of both extraction solvents (55% MeOH and 30% ACN) was further evaluated with the respective analytical validations of the EIA kit by assessing precision, specificity, the lower limit of quantification, and accuracy. Extracts of two of the pools (Pools 1 and 4) used in the efficiency of extraction procedure were combined to obtain a sole pool for all the validation tests.

#### 2.4.1. Precision

To evaluate the repeatability of the assay, the intra-assay coefficient of variation (CV) was calculated from samples read in duplicate within the same plate (*n* = 15 samples). These samples represent a range of OT concentrations across the standard curve (approximately 40 to 1800 pg/mL), falling within the optimal 20–80% binding range of the assay to ensure reliable quantification.

#### 2.4.2. Specificity

Specificity was assessed with the parallelism and linearity of dilution tests. For the parallelism test, the sample pool was serially diluted two-fold between 1:1 and 1:128 in the assay buffer solution provided by the kit, and each dilution was read in duplicate on the plate. A graph was plotted as the expected OT concentrations for each extraction solvent or standards as a function of the proportion binding (B/B0). Only those dilutions that fell within the standard curve limits (neat to 1:16) were used to generate the curve. Results were assessed by visual inspection of the respective curves generated to verify parallel displacement with the standard curve.

Linearity of dilution was determined by diluting the sample pool at multiple ratios (neat, 1:2, 1:4, 1:8, 1:10, and 1:16) with the kit assay buffer. The correlation between the expected and the observed fOT concentrations for each extraction solvent was analyzed using the Pearson’s Product correlation test.

#### 2.4.3. Lower Limit of Quantification

The lower limit of quantification of the assay was determined from the lowest amount of hormone concentration analyzed with acceptable levels of precision and accuracy. The sample pool was diluted at multiple ratios (neat to 1:128) and samples were read in duplicate. To determine precision, the CV for the average concentration of each fOT dilution was calculated. Accuracy was measured by calculating the CV between the expected and the observed fOT concentrations. The low limit of quantification was determined by identifying the lowest OT dilution in which all the CVs were <20%.

#### 2.4.4. Accuracy

Accuracy was assessed through the spike-and-recovery test by spiking 60, 150, or 240 µL of three OT standard solutions provided by the assay kit (102.4 pg/mL, 640 pg/mL, and 1600 pg/mL) into known volumes (60, 150, or 240 µL) of the sample pool (MeOH: 390 pg fOT/mL; ACN: 420 pg fOT/mL). For each combination, the recovery percentage was calculated from the observed and expected fOT concentrations. Additionally, the correlation between the expected and the observed fOT concentrations for each extraction solvent was analyzed using the Pearson’s Product correlation test.

### 2.5. Quantification of fOT in Lions and Relationship with fGM Levels

Based on the results, 55% MeOH was determined to be the most suitable solvent for fOT extraction (see Section 3.3). This allowed for the second part of this study (i.e., exploring fOT concentrations in lions) to be performed on extracts obtained in a previous study exploring fecal steroid hormones in captive lions [44]. These extracts had remained in storage at −20 °C for one (BPV, CA, JZ, and MAD extracts) or four years (BCN extracts). These fecal extracts were diluted 1:6 with the assay buffer provided by the EIA kit to measure fOT concentrations. Two pools of samples were read in duplicate in each EIA kit to calculate the inter-assay CV. Additionally, we included the fGM concentrations measured by EIA in the aforementioned study to evaluate the relationship between fOT and fGM in lions.

### 2.6. Statistical Analysis

A statistical analysis of the data and graphical representation were conducted with R software (R-project, Version 4.3.0, R Development Core Team, University of Auckland, New Zealand) and Graph Pad Software Inc. (GraphPad Prism, version 8.0.2; Graph Pad Software Inc., San Diego, CA, USA), respectively. The statistically significant level was settled at a *p* value < 0.05 in all tests performed.

Individual peak and baseline fOT concentrations were identified following an iterative process in which all concentrations greater than the mean + 1.5 standard deviations (SD) were systematically eliminated until no values exceeded that cut-off (mean + 1.5 SD) to determine the subject’s baseline. All values exceeding the cut-off were classified as peaks.

Normal distribution of the data was assessed by visual inspection of histograms and QQ plots and through the Shapiro–Wilk normality test. Data was log-transformed to achieve normality. To explore differences in fOT concentrations, we used generalized linear mixed models (GLMM) with Gaussian distribution and identity link function. A first model evaluated the effect of age, sex, and their interaction on the overall or baseline fOT concentrations, including zoo location and the individual as random effects. A second model assessed the influence of the female’s contraceptive status (i.e., intact, contracepted, and ovariectomized females) on the overall or baseline fOT levels, with zoo location and individual as random effects. A third model was built to evaluate the effect of zoo location on the overall or baseline fOT levels, including the individual as a random factor. Normal distribution and homogeneity of the residuals was verified by visual inspection of the residuals against fitted values plot and a QQ plot of the residuals. Wald’s χ^2^ tests were employed to evaluate the marginal significance of the fixed factors for each model and, when statistically significant, Tukey-adjusted post hoc pairwise comparisons were conducted.

Spearman’s rank correlation tests of untransformed data were used to assess the relationship between fOT and FGM concentrations for all samples combined, for individual mean concentrations, and for the data of each individual separately.

## 3. Results

### 3.1. Extraction Efficiency for the Two Solvents

The recovery percentages for the extraction efficiency procedure are shown in Table 1.

### 3.2. Assay Validation

#### 3.2.1. Precision

The intra-assay CV (mean ± SD) was 5.90 ± 4.26% for the samples extracted with 55% MeOH and 8.95 ± 8.43% for the samples extracted with 30% ACN.

#### 3.2.2. Specificity

**Figure 1:**
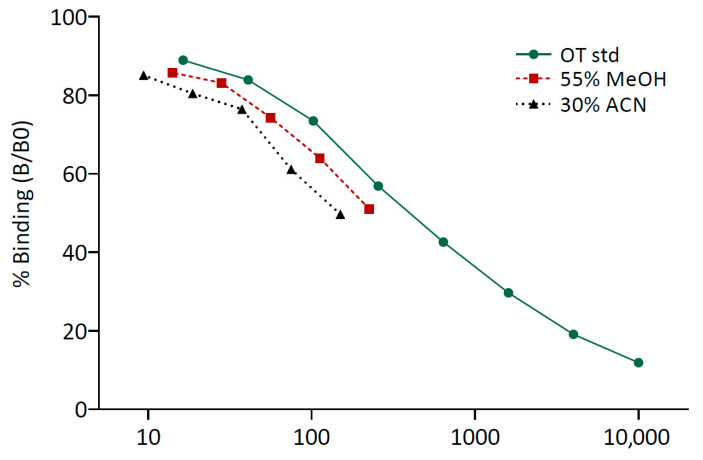
Parallelism between the oxytocin standard curve (OT std) and the curves generated by serial dilutions of the pools of feces extracted with 55% methanol (MeOH) and 30% acetonitrile (ACN). An arbitrary concentration was chosen for the neat sample of each fecal pool to better visualize the parallel displacement with the standard curve.

Serial dilutions of the pool extracted with 55% MeOH and the pool extracted with 30% ACN both showed parallel displacement with the standard curve (Figure 1). In the linearity of dilution test, the obtained fOT concentrations strongly correlated with the expected values for both extraction solvents (Pearson’s correlations: R^2^ = 0.99, *p* < 0.001 and R^2^ = 0.96, *p* < 0.01 for 55% MeOH and 30% ACN, respectively).

#### 3.2.3. Lower Limit of Quantification

The lower limits of quantification were 30.39 ng fOT/mL for the pool of feces extracted with 55% MeOH and 11.08 ng fOT/mL for the pool extracted with 30% ACN.

#### 3.2.4. Accuracy

In the spike-and-recovery tests, the mean (±SD) recovery percentages were 113.47 ± 11.41% and 105.70 ± 13.70% for the pool of feces extracted with 55% methanol (MeOH) and 30% acetonitrile (ACN), respectively. The obtained fOT concentrations strongly correlated with the expected values for both extraction solvents (Pearson’s correlations: R^2^ = 0.98 and 0.96, *p* < 0.001 for 55% MeOH and 30% ACN, respectively).

### 3.3. Selection of the Extraction Solvent

Both solvents were equally efficient in extracting fOT from fecal samples, although the pool samples extracted with 30% ACN showed greater variability in %REE than those extracted with 55% MeOH (Table 1). Additionally, all the parameters of the assay validation were within acceptable ranges for both extraction solvents. Based on these results, and given that steroid hormones are usually extracted with methanol, we selected 55% MeOH as the solvent for the extraction of fOT from feces. The selection of this solvent implied simplifying the laboratory methodology, reducing the use of consumable supplies and chemical reagents, thus optimizing resources and being more time efficient. Therefore, data presented hereinafter were extracted with 55% MeOH as previously described.

### 3.4. fOT Concentrations in Captive Lions

Fecal OT levels were above the lower limit of quantification of the assay in all but one the fecal samples analyzed (1 out of 162 fecal samples). The inter-assay CV was 5.09 ± 0.88%. Concentrations ranged from 3.00 to 296.64 ng fOT/g of feces, with high variability observed within and between individuals (Table 2).

**Figure 2:**
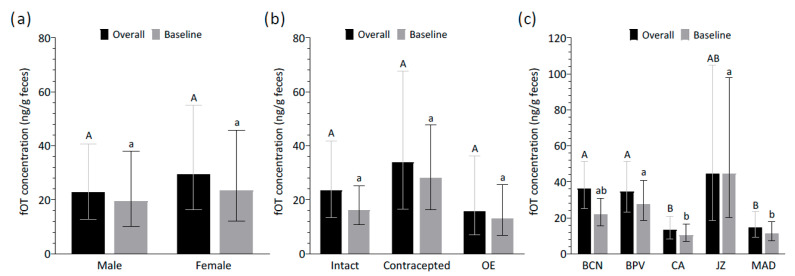
Effect of (**a**) sex, (**b**) contraceptive status in females, and (**c**) zoo location on overall and baseline fecal oxytocin (fOT) concentrations. Data represents back-transformed least-square means with upper and lower asymptotic confidence limits. Different upper- and lower-case letters represent significant differences (*p* < 0.05) between groups in the overall and baseline fOT concentrations, respectively. Abbreviations: OE, ovariectomized; BCN, Barcelona Zoo; BPV, Bioparc Valencia; CA, Parque de la Naturaleza de Cabárceno; JZ, Zoobotánico Jerez; MAD, Zoo Aquarium de Madrid.

Age had no significant influence on the overall and baseline fOT concentrations (χ^2^ = 2.59, df = 1, *p* > 0.05 and χ^2^ = 2.19, df = 1, *p* > 0.05, respectively). Furthermore, the fOT levels did not vary across sexes (overall: χ^2^ = 0.85, df = 1, *p* > 0.05; baseline: χ^2^ = 0.35, df = 1, *p* > 0.05) (Figure 2a). The interaction between sex and age was also assessed in the models and was not significant for either the overall or baseline fOT concentrations (overall: χ^2^ = 0.16, df = 1, *p* > 0.05; baseline: χ^2^ = 0.45, df = 1, *p* > 0.05). Among females, the fOT levels did not differ across intact, contracepted, and ovariectomized lionesses, thus indicating no significant effect of contraceptive status on the overall and baseline fOT levels (χ^2^ = 1.79, df = 2, *p* > 0.05 and χ^2^ = 3.61, df = 2, *p* > 0.05, respectively) (Figure 2b).

Significant differences in the overall and baseline fOT concentrations were found across zoos (overall: χ^2^ = 20.50, df = 4, *p* < 0.001; baseline: χ^2^ = 20.00, df = 4, *p* < 0.001). Post hoc pairwise comparisons revealed that, for overall concentrations, CA and MAD had significantly lower values than BCN and BPV (*p* < 0.05). Likewise, for baseline concentrations, CA and MAD again showed lower fOT levels than BPV and JZ (*p* < 0.05) (Figure 2c).

### 3.5. Relationship Between fOT and fGM Concentrations in Lions

**Figure 3:**
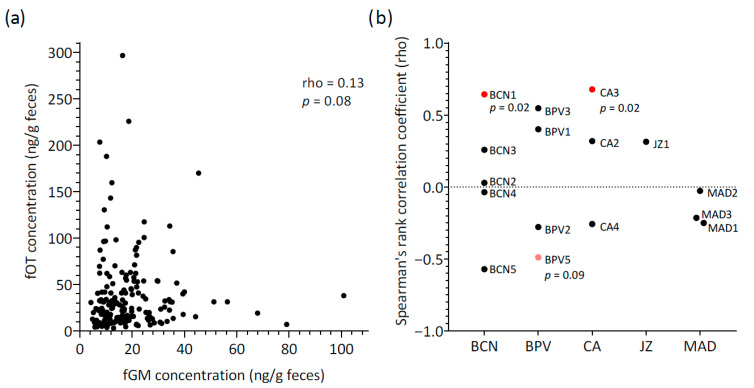
Relationship between fecal oxytocin (fOT) and fecal glucocorticoid metabolite (fGM) concentrations: (**a**) Scatter plot for all samples (*n* = 162) showing the correlation between fOT and fGM (Spearman’s rank correlation test); (**b**) Results of the individual Spearman’s rank correlation tests conducted separately for each lion. Colors indicate the significance level of each individual’s correlation: red, statistically significant (*p* < 0.05); pink, trend towards significance (*p* < 0.1); black, no significant correlation. Abbreviations: BCN, Barcelona Zoo; BPV, Bioparc Valencia; CA, Parque de la Naturaleza de Cabárceno; JZ, Zoobotánico Jerez; MAD, Zoo Aquarium de Madrid; *p*, *p* value (shown only for trends (*p* < 0.1) and statistically significant results (*p* < 0.05)).

Fecal OT concentrations did not correlate with fGM levels for all samples combined (rho = 0.13, *p* = 0.08, *n* = 162) (Figure 3a). When considering mean concentrations across individuals, a moderate positive correlation between hormones was detected between hormones, with the result approaching statistical significance (rho = 0.50, *p* = 0.05, N = 16 lions).

**Figure 4:**
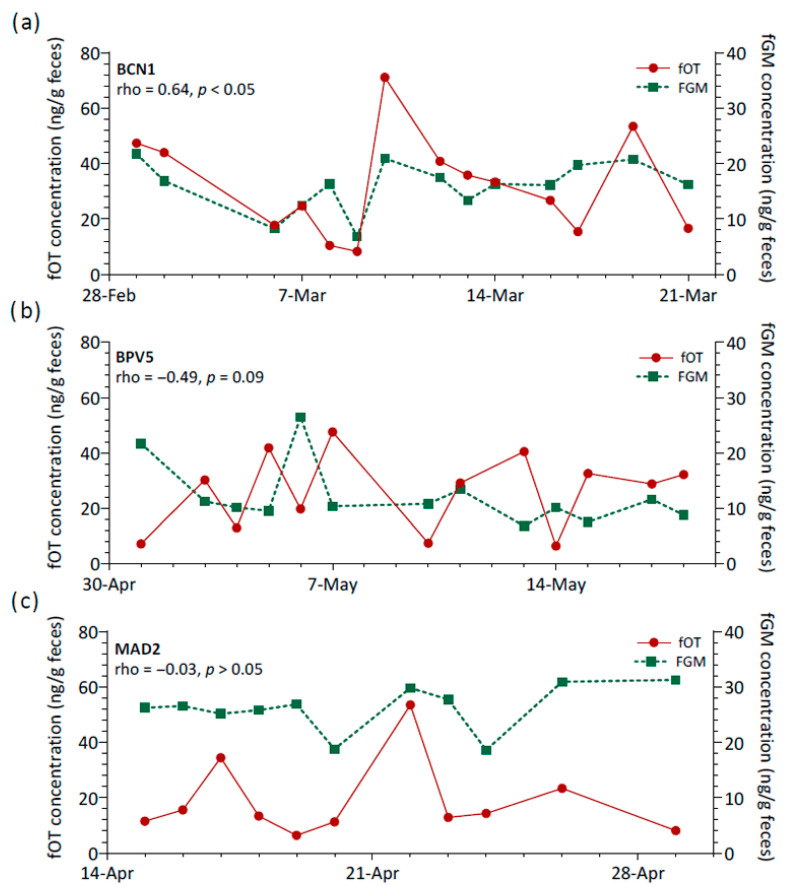
Relationship between fecal oxytocin (fOT) and fecal glucocorticoid metabolites (fGM) concentrations across time for three lions (BCN1, BPV5 and MAD2) as examples of (**a**) a strong and significant positive correlation, (**b**) a moderate, though not significant, negative correlation, and (**c**) a virtually negligible correlation. Abbreviations: rho, Spearman’s rank correlation coefficient; *p*, *p* value.

Additionally, the relationship between fOT and fGM concentrations was assessed longitudinally for each lion, and marked variability was observed (Figure 3b). Spearman’s rank coefficients of correlation were generally negligible to moderate and non-significant (rho range = −0.57 to 0.55, *p* > 0.05, N = 13 lions). Only two lions showed a significant positive correlation (rho = 0.64 (BCN1) and 0.68 (CA3), *p* < 0.05), and a third lion (BPV5) presented a tendency towards significance for a negative correlation between fOT and fGM (rho = −0.49, *p* = 0.09). Therefore, the three main patterns of variation observed for fOT and fGM levels over time consisted of changes in the concentrations of both hormones generally in a reciprocal manner (Figure 4a), concentrations varying apparently in opposite directions (Figure 4b) or with no apparent inter-relationship between fOT and fGM levels (Figure 4c).

## 4. Discussion

This study is the first to publish the use of feces for measuring peripheral OT, using lions as a model species. Our findings suggest that the developed methodology is effective for extracting OT from feces, and that fOT can be consistently detected in lions using a commercially available EIA. In addition, we explored how fOT concentrations vary among captive lions under baseline housing and husbandry conditions. We found marked variability in the fOT levels both within and between individuals, as well as across zoos. Notably, in this study, no significant relationships were found between the fOT levels and factors such as age, sex, and contraceptive status in females. Furthermore, no consistent or clear relationship between fOT and fGM was observed under baseline conditions in the lions.

### 4.1. Development of a Reliable Methodology for the Measurement of OT in Feces

The methodology employed for extracting fOT was adapted from protocols commonly used for fecal steroid hormone extraction [37,46]. Although solid phase extraction is often applied for purifying and concentrating analytes, like steroids and peptides, from complex matrices [22], this approach can be complex, costly and time-consuming [46]. This makes it unfeasible for some laboratories, especially those working with limited resources or conducting large-scale, longitudinal studies. By contrast, we developed a simple solvent extraction protocol that offers an accessible and efficient alternative for monitoring fOT in wild species. Both organic solvents tested (55% MeOH and 30% ACN) were equally efficient at extracting OT from feces and performed well in analytical validation of the assay, although greater variability in %REE was observed for 30% ACN. Hence, the decision to select 55% MeOH as the extraction solvent for the second part of this study was mostly based on practical considerations, reducing time and costs in the lab. A previous study by López-Arjona et al. [32] also applied a steroid hormone-based extraction protocol for OT extraction from pig hair, and evaluated the OT extraction efficiency of MeOH and ACN. Unlike our findings, they reported that MeOH was significantly more efficient than ACN in recovering OT from hair. These differing results may be explained by the percentage of organic solvent employed in each study. While López-Arjona et al. used pure solutions (100% MeOH and 100% ACN), we optimized solvent percentages based on Gnanadesikan et al. [50] for a more selective OT extraction, maximizing OT recovery without increasing potential sources of interference.

A careful analytical validation of the assay was conducted for the measurement of OT in feces in lions using MeOH and ACN extraction. The Arbor Assay kit performed well in all the analytical validation tests, suggesting that OT can be consistently detected in lion feces. However, given that immunoassays rely on antibody binding to specific regions of the target analyte, structurally similar molecules may cross-react with the assay antibody and generate a false-positive signal [23]. In accordance, Gnanadesikan et al. [50] suggested that the Arbor Assays anti-OT antibody may cross-react with potential OT metabolites, but also with other unrelated metabolites of similar structure, and thus emphasize how optimal extraction methods are essential to minimize interference.

While we present a first approach for measuring OT in feces, several methodological issues have yet to be investigated. For instance, it is well-known that hormone concentrations may be impacted by the way samples are handled prior to analysis [51], and matrices such as urine and feces are particularly prone to degradation due to the presence of bacteria [52]. Accordingly, one potential limitation in our study may be related to fOT stability, as the samples used for the second part of this study (i.e., exploring fOT concentrations in lions) had been previously extracted and stored at −20 °C for several years before fOT quantification. Given that the samples were not freshly extracted, we cannot be certain whether the concentrations measured truly reflect the original fOT levels or were affected by some potential degradation over time. However, for samples stored for four years (BCN), as shown in Table 2, the individual mean concentrations, ranges, and CVs were comparable to those of lions from other zoos, whose samples had been stored for only one year before analysis. Furthermore, statistical comparisons across zoos (Figure 2c) did not reveal systematic differences in the fOT concentrations between BCN and the other zoos. Altogether, these findings suggest that extended storage time in BCN samples did not result in consistently higher or lower values, supporting the idea that degradation over time was unlikely to have had a major impact on the results. Studies on urinary OT in humans, dogs, and wolves have shown that, while concentrations increase with longer storage durations, OT levels remain most stable when stored as urine extracts at −20 °C [25,52]. Given the absence of comparable data in feces, future studies should investigate the stability of fOT under varying sample handling and storage conditions.

In addition, since fOT has not been previously investigated, the pathways through which this peptide might appear in feces, and whether the parent molecule, OT-derived metabolites or both are detected, remains unknown. As any peptide hormone, OT is degraded via proteolysis into smaller, sometimes biologically active, fragments by specific enzymes in the bloodstream and organs, such as the liver and kidneys [1,53]. Beyond this, the finding that OT is also locally produced and secreted from enterocytes in the intestinal epithelium [15] opens a new set of possibilities for understanding the potential sources of OT in feces. As happens with OT produced in other peripheral tissues, the specific roles of local OT in the gastrointestinal tract are not fully understood. Current research suggests that enterocytic OT might be involved in the regulation of gut motility and food metabolism in a species-specific manner, though less is known on its potential effects beyond the gut [15,16,54]. Nevertheless, regardless of the source of origin, the key question remains whether the measured fOT is reflective of central oxytocinergic activity, including its role in mediating stress responses and social behavior.

### 4.2. Exploring the fOT Levels in Captive Lions

Our findings revealed substantial variability in the fOT levels both within and between lions. Similarly, marked interindividual variation was reported in salivary OT levels in an all-males group of Asian elephants (*Elephas maximus*) [55], and in urinary OT concentrations in tamarins (*Saguinus oedipus*) [56]. Research in humans and lab rodents has attributed individual differences in OT concentrations, and in the responsiveness of the oxytocinergic system, to several biological factors (i.e., age, sex, and genetics) and environmental influences (e.g., early life experiences) [57]. However, the impact of such factors on endogenous OT concentrations in domestic and wild animal species remains underexplored.

Some studies have provided a first insight into the relationship between age and peripheral OT levels in wildlife species. The research in bottlenose dolphins (*Tursiops truncatus*) [58], western lowland gorillas [59], and rhesus macaques (*Macaca mulatta*) [60] suggests that immature individuals may present higher peripheral OT levels compared to older age groups, where no significant age-related differences have been found. Consistent with these findings, the fOT levels did not differ with age in the adult lions studied, supporting the idea that, although age may influence peripheral OT levels, this effect appears to be restricted to younger individuals. Likewise, no significant age- or sex-related effects on urinary OT levels have been observed in response to social interactions in wild chimpanzees [28,61,62].

In this study, concentrations of fOT were similar between males and females. Interestingly, although the limited sample size warrants cautious interpretation, similar patterns have been reported in urinary OT in domestic cats [63,64] and in plasma OT in bottlenose dolphins [58], where no sex differences were described. However, surprisingly, the fOT levels did not differ across the lionesses’ contraceptive status (i.e., intact, hormonally contracepted, and ovariectomized). This was unexpected because estrogen is thought to play an important role in regulating OT [27,65], and non-cycling females have reduced estrogen production. Nevertheless, these findings should be interpreted with caution due to the limited sample size per individual and the potential influence of interindividual variability and hormone secretion patterns. Unlike our results, Snowdon et al. [56] demonstrated that estrogen treatment resulted in increased endogenous (urinary) OT in male tamarins. Similarly, other wildlife studies have also shown that the peripheral OT levels can vary with female reproductive status, such as in female baboons during the periovulatory period [27] and in lactating grey seal (*Halichoerus grypus*) mothers [66], although this pattern was not observed in lactating bottlenose dolphin mothers [58]. In our study, several factors may have contributed to the lack of differences in the fOT levels between contraceptive groups. For instance, we could not confirm whether the intact females were actively cycling at the time of sampling, nor whether they were at the same stage of the ovarian cycle, which may have contributed to the observed interindividual variability. Additionally, the relatively advanced age of most females may have influenced their reproductive physiology and hormone secretion. Zoos could also be a potential confounding factor since, except for hormonally contracepted females, each zoo included only one contraceptive status.

Fecal OT levels also varied between zoo locations, with CA and MAD presenting lower concentrations than the other zoos. Although the underlying causes are unknown, differences in housing conditions and management practices, as well as social structure, most likely played a part. The variability in endogenous OT levels has been investigated in other wild species in response to several housing and husbandry conditions and their social environment. For instance, urinary OT levels were evaluated in zoo-housed male gorillas, revealing that males in bachelor groups had higher OT levels than those in mixed-sex groups or singly housed, thus suggesting stronger affiliative relationships in bachelor groups [59]. Additionally, the urinary OT concentrations in a male gorilla significantly decreased following the death of his group mate [19]. Similarly, the urinary OT levels increased in pair-bonded tamarins when changing from solitary housing to being paired with a mate [56]. Yet, in adult male rhesus macaques, the plasma OT levels did not differ between single-housed and highly affiliative pair-housed conspecifics [60]. To our knowledge, only one study has evaluated the effects of OT on sociability in lions, demonstrating that intranasal OT administration increased social proximity among group members and reduced vigilance toward out-group stimuli [67].

### 4.3. Relationship Between fOT and fGM Concentrations in Lions

Although different analytical approaches yielded varying results, we found no consistent correlation between fOT and fGM levels. The complex interplay between the oxytocinergic system and the HPA axis is not fully understood and may differ between basal and stressful contexts. In response to stressors, evidence shows that OT can down-regulate the activity of the HPA axis, facilitating the return to baseline GC concentrations [7,68,69]. However, this stress-induced release of OT is not always consistent within the same stressor and may be context specific (see [70]). While most studies assess the relationship between both systems in response to stressful events or challenges, much less is known on the relationship between OT and GCs under basal (non-stressful) conditions. In this regard, a meta-analysis in humans found only a negligible positive correlation between peripheral OT and cortisol levels under baseline conditions (Pearson r = 0.16, *p* = 0.008), with considerable variability across studies [71].

The adult lions in our study were housed in socially stable groups and under basal environmental and husbandry conditions, with no acute or unusual stressors introduced during the sampling period beyond the everyday stimuli of a zoo setting. While no correlation was observed between fOT and fGM levels within samples, we did find a moderate positive correlation at the threshold for statistical significance when comparing mean concentrations across the lions. However, applying a longitudinal approach to assess this relationship independently for each lion revealed no consistent pattern across individuals, with considerable variability in both the strength and direction of the relationship between hormones. Previous studies exploring the relationship between peripheral (urinary) OT and GCs under baseline conditions in other species have also reported mixed results. For example, while a moderate positive correlation approaching statistical significance (Spearman R_s_ = 0.48; *p* = 0.07) was observed in shelter cats [64], no significant correlation was found in household cats [63]. Similarly, urinary OT and GCs were significantly positively correlated in grey wolves, but this relationship did not hold for dogs within the same study [72].

The lack of a clear pattern of correlation between fOT and fGM in lions in the present study may suggest that both systems function independently under typical basal conditions for the species. Nevertheless, we cannot rule out the possibility that, if fOT originates from local production in the gut, its relationship with the HPA axis may differ from the hormone patterns observed in other biological matrices. Future investigations should focus on evaluating how fOT fluctuates in response to known stressors to shed light on the interplay between the oxytocinergic system and the HPA axis activity under stressful conditions for this new matrix.

### 4.4. Limitations and Future Directions

While our study constitutes a fundamental first step in the development of a reliable method to measure OT in feces, some limitations must be acknowledged. As mentioned, methodological aspects still need to be evaluated, including the stability of fOT under various sampling and storage conditions. More importantly, whether the immunoreactivity detected by the assay kit is specific to OT, its metabolites, or other potentially interfering substances remains unknown. Therefore, future studies should employ more advanced characterization techniques, such as high-performance liquid chromatography and mass spectrometry, to confirm the presence of the OT molecule in feces and to identify the source of the detected immunoreactivity.

Another major limitation is the lack of a robust biological validation demonstrating that fOT reflects biologically meaningful changes and, therefore, that it can be a useful welfare biomarker in behavioral and stress-related studies. To move forward, it is important to conduct studies in more controlled settings where endogenous OT levels are expected to vary, such as following positive social interactions (e.g., affiliative behavior or mother–offspring contact), exposure to stressors that may activate the oxytocinergic system, or after pharmacological administration of OT or other agents known to influence its release. Additionally, serial sampling over defined time periods following such stimuli could help clarify excretion patterns and improve the interpretability of fOT measurements.

In addition, although we present a multi-centric study including samples from five zoological institutions, the sample size is still relatively small, thus findings on the basal fOT patterns in captive lions should be interpreted with caution. Future research should thus focus on addressing all these limitations and prioritize performing a robust biological validation that measures fOT, along with other validated behavioral and physiological metrics, such as GCs, in well-defined contexts known to elicit endogenous OT release.

## 5. Conclusions

This study provides a foundational methodology for extracting and quantifying OT from feces, demonstrating that fOT can be consistently detected in lions. While substantial variability in the fOT levels was observed across individuals and zoos, we did not detect significant effects of age, sex, or reproductive status in this study. Additionally, no consistent relationship was found between fOT and fGM under baseline conditions. While these findings offer preliminary insights into the dynamics of fOT in captive lions, this study represents an important first step toward establishing a novel non-invasive tool for investigating OT in various social and environmental contexts. Future research should focus on refining the methodology and validating fOT as a biomarker of animal welfare across diverse species.

## Figures and Tables

**Table 1:** Results of the extraction efficiency procedure indicating the recovery percentage (%REE) for the two extraction solvents evaluated (55% MeOH and 30% ACN). Concentrations of the spiked oxytocin (OT) standards and observed OT concentrations in the fecal pool samples (Pools 1 and 2: females; Pools 3 and 4: males) are also shown.

Extraction Solvent	Spiked OT Standard (pg/mL)	Sample	Observed OT Concentration (pg/mL)	%REE	%REE (Mean ± SD)	Global %REE (Mean ± SD)
55% MeOH	820 (high OT)	Pool 1	920.52	90.64	101.12 ± 15.29	95.45 ± 13.25
		Pool 2	930.54	107.87		
		Pool 3	788.23	86.57		
		Pool 4	1204.97	119.40		
	370 (low OT)	Pool 1	509.04	90.21	89.79 ± 9.49	
		Pool 2	420.14	101.75		
		Pool 3	367.30	78.58		
		Pool 4	551.55	88.62		
30% ACN	820 (high OT)	Pool 1	763.33	70.79	116.01 ± 30.49	99.31 ± 28.23
		Pool 2	1087.18	124.72		
		Pool 3	1184.52	133.50		
		Pool 4	1323.71	135.03		
	370 (low OT)	Pool 1	432.32	67.84	82.62 ± 13.65	
		Pool 2	360.09	80.47		
		Pool 3	460.71	100.91		
		Pool 4	514.87	81.26		

Abbreviations: MeOH, methanol; ACN, acetonitrile.

**Table 2:** Biological data of the studied lions and descriptive statistics of fecal oxytocin levels (fOT; ng/g feces) including sample size (*n*), mean (±SD) overall fOT concentrations, range, coefficient of variation (CV), and proportion of peaks calculated through the iterative process.

Lion ^a^	Age	Sex	Contraceptive Status ^b^	*n*	Mean ± SD	Range	CV (%)	Peaks (%)
BCN1	3	M	Intact	14	31.87 ± 18.17	8.30–71.16	57	50
BCN2	15	M	Intact	6	90.91 ± 85.49	22.36–225.81	94	33
BCN3	15	F	Contracepted	12	49.91 ± 53.94	8.86–203.36	108	33
BCN4	15	F	Contracepted	11	43.82 ± 37.08	6.56–117.46	85	45
BCN5	15	F	Contracepted	7	56.15 ± 50.15	15.65–159.66	89	14
BPV1	16	F	Ovariectomized	13	73.87 ± 55.95	10.85–187.99	76	23
BPV2	5	M	Intact	15	42.87 ± 23.31	13.39–98.14	54	40
BPV3	6	F	Ovariectomized	8	41.34 ± 27.43	12.67–96.31	66	13
BPV5	10	F	Ovariectomized	13	25.88 ± 13.95	6.43–47.60	54	8
CA2	6	M	Intact	14	10.72 ± 5.97	4.17–22.95	56	36
CA3	13	F	Intact	12	25.28 ± 29.30	3.00–112.90	116	25
CA4	13	F	Intact	6	18.38 ± 10.90	4.41–33.94	59	0
JZ1	8	M	Intact	6	52.96 ± 33.27	16.54–100.55	63	0
MAD1	7	F	Contracepted	7	9.60 ± 3.50	3.75–15.36	36	14
MAD2	11	M	Intact	11	18.63 ± 13.95	6.41–53.57	75	18
MAD3	7	F	Contracepted	7	58.12 ± 105.69	5.67–296.64	182	14

Abbreviations: BCN, Barcelona Zoo; BPV, Bioparc Valencia; CA, Parque de la Naturaleza de Cabárceno; JZ, Zoobotánico Jerez; MAD, Zoo Aquarium de Madrid; F, female; M, male; SD, standard deviation. ^a^ Two females (BPV4 and CA1) were excluded from this study for not meeting the established criteria. Female BPV4 was diagnosed with a reproductive disease and CA1 was being segregated from the group. ^b^ Contraception consisted of deslorelin acetate implants (SUPRELORIN^®^, Virbac, France).

## Data Availability

The original contributions presented in this study are included in the Supplementary Material. Further inquiries can be directed to the corresponding author(s).

## Supplementary Materials

The following supporting information can be downloaded at: https://www.mdpi.com/article/10.3390/ani15162409/s1: Raw dataset containing all original measurements of fOT and fGM concentrations and associated metadata used in this study.
